# Anomalous origin of the coronary artery: prevalence and coronary artery disease in adults undergoing coronary tomographic angiography

**DOI:** 10.1186/s12872-024-03942-8

**Published:** 2024-05-23

**Authors:** Kunyan Li, Ping Hu, Xiaolin Luo, Furong Li, Ling Chen, Junyong Zhao, Zelan Wang, Wenjian Luo, Jun Jin, Zhexue Qin

**Affiliations:** 1 Department of Cardiology, Xinqiao Hospital, Army Medical University, Shapingba District, Chongqing, China; 2 Department of Neurology, the First Affiliated Hospital of Chongqing Medical University, Chongqing, China; 3 Department of Nephrology, Xinqiao Hospital, Army Medical University, Shapingba District, Chongqing, China

**Keywords:** Anomalous aortic origin of coronary artery, Coronary artery disease, Coronary computed tomographic angiography

## Abstract

**Background:**

Anomalous aortic origin of a coronary artery (AAOCA) is a rare congenital coronary anomaly with the potential to cause adverse cardiac events. However, there is limited data on the association between AAOCA and coronary artery disease (CAD). Therefore, the aim of this study is to determine the prevalence and symptoms of patients with AAOCA, as well as investigate the correlation between AAOCA and CAD in a population referred for coronary computed tomographic angiography (CTA).

**Methods and results:**

All consecutive patients who underwent CTA from 2010 to 2021 were included. Characteristics, symptoms, coronary related adverse events and CTA information were reviewed by medical records. Separate multivariable cumulative logistic regressions were performed, using the stenosis severity in each of the four coronaries as individual responses and as a combined patient clustered response. Finally, we identified 207 adult patients with AAOCA, the prevalence of AAOCA is 0.23% (207/90,501). Moreover, this study found no significant association between AAOCA and CAD. AAOCA did not contribute to higher rates of hospitalization or adverse cardiac events, including calcification.

**Conclusion:**

AAOCA is a rare congenital disease that is not associated with increased presence of obstructive CAD in adults.

**Supplementary Information:**

The online version contains supplementary material available at 10.1186/s12872-024-03942-8.

## Introduction

Anomalous aortic origin of coronary artery (AAOCA) is a congenital malformation whereby a coronary artery originates aberrantly, such as from an opposite sinus, or above the sinuses of Valsalva. Data from autopsy, echocardiography and coronary studies have shown the prevalence of 0.1–1.0% [1, 2]. Coronary computed tomographic angiography (CTA) is generally used to diagnose and evaluate the coronary paths, including origin and course, degree of luminal narrowing, the relationship to surrounding structures and concomitant obstructive coronary artery disease [3]. It is noted that AAOCA can cause symptoms such as syncope, angina, chest pain and dyspnea [4–7]. Several publications have reported that 37% of sudden death victims experienced premonitory symptoms like chest pain and syncope [5]. However, other researchers argue that an increasing number of patients present with vague symptoms [1, 8–10]. Therefore, there are still ongoing debates regarding the relationship between AAOCA and its associated symptoms.

Previous studies on AAOCA have identified higher frequencies of ischemia and sudden cardiac death (SCD) among specific anatomical variants [11]. Notably, the left coronary artery originating from the right coronary sinus with an inter-arterial course is considered as high-risk [12, 13]. The cumulative risk of SCD for young athletes aged 15–35 years is approximately 6.3% for those with anomalous left main coronary artery (LMCA) and 0.2% for those with anomalous right coronary artery (RCA) [9, 14]. Amado et al. also found that individuals with anomalous aortic origin of a coronary artery featuring LMCA originating from the right sinus of Valsalva face an increased risk of sudden death and adverse cardiac events compared to those with AAOCA featuring RCA originating from the left sinus [15]. The various pathological aspects associated with malignant AAOCA collectively contribute to a significantly reduced functional ostial area in the anomalous coronary artery, leading to ischemia and potentially fatal arrhythmias [16]. The potential mechanisms underlying AAOCA-induced ischemia may be resulted from the origin, the anatomy of the origin [16–19], the angulation, and the dynamic compression [12, 20–22].

There is limited literature exploring the relationship between coronary artery disease (CAD) and AAOCA, and as of now, there is no consensus on this matter. The elevated time-averaged wall shear stress found in AAOCA patients could potentially lead to premature atherosclerosis and coronary spasm [23]. Click et al. reported a significantly greater degree of stenosis in anomalous circumflex arteries compared to normal ones in the control group [24]. Similarly, others found that the degree of stenosis was significantly higher in anomalous vessels than other coronary arteries within the same patient [25]. They suggested that the unusual angle of takeoff and more tortuous course of the proximal portion of the anomalous coronary artery predisposes them to accelerated atherosclerosis [26]. By creating computer aided designed models, Formato et al. showed that intramural course may lead to coronary narrowing, which leads to decreased blood flow and then leads to myocardial ischemia [27]. However, other studies have suggested that there is no association between coronary artery anomaly and CAD [28, 29]. Thus, it remains unclear if AAOCA is associated with development of CAD. The objective of the present study was to determine the prevalence and symptoms of patients with AAOCA, as well as investigate the correlation between AAOCA and CAD in a population referred for CTA at a single center.

## Methods

### Patients

A retrospective analysis of the AAOCA database was used to identify all patients who underwent CTA between April 2010 and May 2021 in Xinqiao hospital. This study was approved by the Medical Ethics Committee of the Second Affiliated Hospital of Army Medical University (No. 2023-009-01), and informed consent was waived in accordance with the Declaration of Helsinki. Patient demographics, presenting symptoms, CTA results, and outcomes were reviewed from medical records. Specifically, chest pain, syncope, dizziness, palpitations, dyspnea, angina, history of hypertension, history of diabetes mellitus, history of dyslipidemia, hospitalization history, surgery history and coronary artery calcification were recorded. Inclusion criteria for AAOCA encompassed adults (age ≥ 18 years) with an anomalous LMCA, left anterior descending (LAD), left circumflex (LCx), or RCA arising from an incorrect sinus of Valsalva or above the sinotubular junction. Patients with other hemodynamically significant congenital cardiac malformations requiring independent surgical intervention and those with anomalous origin from other arteries were excluded. Stenosis severity in each coronary artery was categorized as follows: none (0%), minimal (1–24%), mild (25–49%), moderate (50–69%), severe (70–99%). Obstructive CAD referred to obstructive lesions with ≥ 50% stenosis in the LMCA, RCA, LAD, or LCx coronary artery. Multiple coronary anomalies were defined as having two or more coronary origins anomalous. According to the American and European guidelines [12, 13], abnormalities of left coronary origin from the right sinus were considered high-risk.

Between April 2010 and May 2021, a consecutive cohort of 90,501 patients with known or suspected CAD due to chest pain or other indications such as cardiovascular risk factors or electrocardiogram abnormalities underwent CTA at our institution. Among them, 211 cases (0.23%) were identified with AAOCA. After excluding four patients with specific anomalies, including abnormal pulmonary artery origin and post-coronary artery bypass grafting variations, a total of 207 patients (0.23%) were selected for further assessment.

### Statistics

Continuous variables in this study are presented as mean ± standard deviation while categorical data are described using counts and frequencies. Chi-square test and independent-samples T tests evaluated associations between categorical variables, such as whether the presence of obstructive CAD, or which coronary was anomalous and location of obstructive CAD (none, only in the anomalous coronary, or within normal-origin coronaries). Post-hoc analyses compared each of the 3 subgroups of CAD location with the 2 other subgroups (independently and combined) using the Bonferroni correction for significance. Separate multivariable cumulative logistic regressions were performed, using the stenosis severity in each of the four coronaries as individual responses and as a combined patient clustered response. Covariates were assigned at the coronary or patient level in this multilevel model. First, at the coronary level, we categorized the origin as normal or anomalous. Next, at the patient level, covariates considered included age, sex, comorbidities (diabetes, hypertension, and dyslipidemia) and symptoms (chest pain, syncope, dizziness, palpitations, dyspnea, angina). To account for how the anomalous origin affects the stenosis in the other normal-origin coronaries within a patient, we also included which of the 4 coronaries was anomalous at the patient level. *P* < 0.05 was considered as indicate statistical significance. All analyses were performed with SPSS software, version 20 (SPSS, Chicago, IL, USA).

## Results

### Baseline characteristics of patients

Of 207 patients who underwent computed tomography and then were identified as AAOCA in the four main coronaries, 181 were identified as absent coronary artery disease, and 26 were diagnosed with obstructive coronary artery disease. Among 26 patients diagnosed with obstructive coronary disease, 20 patients had obstructive disease in coronary arteries with normal origin, while in 6 patients, the obstruction involved coronary arteries of abnormal origin. Among the 6 patients with obstructive disease in an anomalous coronary artery, 1 had a moderate ostial stenosis. Additionally, 16 cases (0.02%) out of 90,501 patients were identified with high-risk abnormalities.

Among our all cohort of 207 patients, the majority were male (133/207, 64%), with average age of 58.7 ± 11.6 years at the time of CTA imaging (Table 1). As shown in Table 1, patients with obstructive CAD are significantly older than patients with absent coronary artery disease (63.0 ± 10.9, vs. 58.0 ± 11.6 years, *p* = 0.039). Compared with those without obstructive CAD, the subgroup with obstructive CAD have more hypertension (45/181, 24.9% vs. 10/26, 38.5%, *p* = 0.142), more diabetes mellitus (16/181, 8.8% vs. 5/26, 19.2%, *p* = 0.101), more dyslipidemia (12/181, 6.6% vs. 4/26, 15.4%, *p* = 0.118), more chest pain (52/181, 28.7% vs. 9/26, 34.6%, *p* = 0.538), more angina (5/181, 2.8% vs. 2/26, 7.7%, *p* = 0.193), less dyspnea (6/181, 3.3% vs. 0/26, 0%, *p* = 0.346), and palpitation were almost the same (13/181, 7.2% vs. 2/26, 7.7%, *p* = 0.925), more syncope (2/181, 1.1% vs. 2/26, 7.7%, *p* = 0.023), more dizziness (5/181, 2.8% vs. 4/26, 15.4%, *p* = 0.003) (Table 1). As shown in Table 1, there were statistically significant differences in symptoms of syncope and dizziness between the two groups. Further subgroup analysis showed (Table S1) that the cases of syncope (*n* = 2) and dizziness (*n* = 4) were all from patients with CAD whose coronary stenosis occurred in those coronary arteries of normal origin.

**Table 1:** Demographics, symptom and comorbidities stratified by presence of obstructive CAD

	All	Absent CAD	CAD	*P* value
	*N* = 207	*N* = 181	*N* = 26	
	*n*(%)	*n*(%)	*n*(%)	
Age, years	58.7 ± 11.6	58.0 ± 11.6	63.0 ± 10.9	0.039
Male	133(64.3)	112(61.9)	21(80.8)	0.060
Presenting symptom				
Chest pain	61 (29.5)	52 (28.7)	9(34.6)	0.538
Syncope	4 (1.9)	2 (1.1)	2(7.7)	0.023
Dizziness	9 (4.3)	5 (2.8)	4 (15.4)	0.003
Palpitation	15 (7.2)	13 (7.2)	2 (7.7)	0.925
Dyspnea	6 (2.9)	6 (3.3)	0 (0)	0.346
Angina	7 (3.4)	5 (2.8)	2 (7.7)	0.193
Risk factors				
Hypertension	55 (26.6)	45 (24.9)	10 (38.5)	0.142
Diabetes mellitus	21 (10.1)	16 (8.8)	5 (19.2)	0.101
Dyslipidemia	16 (7.7)	12 (6.6)	4 (15.4)	0.118

Baseline characteristics of 207 adult patients diagnosed with AAOCA are shown as counts (and relative frequencies) stratified by the presence. Obstructive coronary artery disease (CAD) was defined as ≥ 50% stenosis in the left main coronary artery (LMCA), the right coronary artery (RCA), left anterior descending (LAD), or left circumflex (LCx) coronary artery. The frequencies of patients with AAOCA affecting each of the 4 or multiple coronaries were stratified based on the presence of any CAD. *P* value resulted from χ^2^ tests and independent-samples T test comparing patients with no CAD and CAD. AAOCA, anomalous aortic origin of a coronary artery

### Presence and location of obstructive CAD

Table 2 shows the most common anomalous coronary was the RCA (137/207, 66.2%), followed by the LMCA (25/205, 12.1%), multiple coronaries (24/207, 11.6%), LCx (20/207, 9.6%), and LAD (1/207, 0.5%). Similar to the distribution, patients without obstructive coronary artery disease most were the anomalous coronary in RCA (117/181, 64.4%), followed by the LMCA (25/181, 13.8%), multiple coronaries (19/181, 10.5%), LCx (19/181, 10.5%), and LAD (1/181, 0.6%). Of 26 patients with obstructive coronary artery disease, 1 (3.8%) had anomalous origin and coronary stenosis in RCA, 1 (3.8%) had anomalous origin in LCx and coronary stenosis in other coronary artery, 19 (73.1%) had anomalous origin in RCA and stenosis in other coronary artery, and 5 (19.2%) had anomalous origin in multiple coronary arteries and stenosis in these coronary arteries.

**Table 2:** Frequency of anomalous coronary stratified by presence and location of obstructive CAD

Anomalous coronary	All	Absent CAD	Only anomalous coronary with CAD	Normal-origin coronary with CAD
	*N* = 207	*N* = 181	*N* = 6	*N* = 20
LMCA	25 (12.1)	25 (13.8)	0 (0)	0 (0)
LAD	1 (0.5)	1 (0.6)	0 (0)	0 (0)
LCx	20 (9.6)	19 (10.5)	0 (0)	1 (5)
RCA	137 (66.2)	117 (64.6)	1 (16.7)	19 (95)
Multiple	24 (11.6)	19 (10.5)	5 (83.3)	0 (0)

The presence of obstructive coronary artery disease (CAD) was defined as obstructive lesions with ≥ 50% stenosis in the left main coronary artery (LMCA), the right coronary artery (RCA), left anterior descending (LAD), or left circumflex (LCx) coronary artery. The frequencies of patients with AAOCA affecting each of the 4 or multiple coronaries were stratified based on the presence and location of any CAD. Patients with obstructive CAD only in the anomalous coronary were compared with those without any CAD and those with CAD in normal-origin vessels. AAOCA, Anomalous aortic origin of a coronary artery

### CAD severity in coronary arteries with normal versus anomalous origin

Among the 4 major coronaries in the 207 patients with AAOCA, 828 coronary arteries had the severity of CAD graded. In the left coronary arteries, 422 (77%) with normal origin is completely stenosis-free, 35 (6.4%) had minimal stenosis, 53 (9.7%) had mild stenosis, 22 (4%) had moderate stenosis, 13 (2.4%) had severe stenosis. The distribution of CAD severity was similar among the right and left coronaries with anomalous origins and those without anomalous origins (Table 3).

**Table 3:** Counts and proportions of origin type with stenosis grade

CAD severity	Left coronary origin		Right coronary origin	
	Normal	Anomalous	Normal	Anomalous
None	422 (77.4%)	67 (88.2%)	48 (92.3%)	114 (73.5%)
Minimal	35 (6.4%)	3 (3.9%)	0 (0%)	17 (11.0%)
Mild	53 (9.7%)	3 (3.9%)	3 (5.8%)	16 (10.3%)
Moderate	22 (4.0%)	2 (2.6%)	0 (0%)	7 (4.5%)
Severe	13 (2.4%)	1 (1.3%)	1 (1.9%)	1 (0.6%)

The proportions of anomalous and normal-origins left and right coronaries with each stenosis severity. The stenosis in coronary arteries with normal origins was similar to those with anomalous origins

Multivariable modeling also showed no association between the presence of anomalous origin and coronary arteries stenosis severity in the corresponding coronary (all *p* > 0.05). Meanwhile, older age (*p* < 0.01), the occurrence of dizziness (*p* < 0.01) were associated with increased stenosis within the 4 coronaries (Table 4). Similar associations were seen with the CAD severity when each of the four coronaries was analyzed as separate response variables. The corresponding odds ratio for increased likelihood of greater CAD severity in all coronaries was 0.9 (95% confidence interval, 0.3–2.5), indicating that LMCA with anomalous origin is not related with stenosis severity. The same correlation was found in other coronary arteries (Table S2-S5). These results suggested that abnormalities of coronary origin were not associated with the severity of CAD (Table 5).

**Table 4:** Factors associated with greater severity of coronary artery stenosis

	Coefficient ± SE	*P* value
Anomalous coronary: LMCA	-0.153 ± 0.594	0.796
Anomalous coronary: LAD	-17.510 ± 0.000	-
Anomalous coronary: LCX	-0.012 ± 0.653	0.985
Anomalous coronary: RCA	-0.149 ± 0.465	0.749
Age, years	0.070 ± 0.015	< 0.000
Gender: male	0.208 ± 0.319	0.513
Chest pain	0.064 ± 0.356	0.857
Syncope	0.371 ± 1.010	0.713
Dizziness	1.735 ± 0.668	0.009
Palpitation	-0.662 ± 0.643	0.303
Dyspnea	-0.222 ± 1.098	0.840
Angina	1.278 ± 0.721	0.076
Hypertension	0.319 ± 0.340	0.347
Diabetes mellitus	0.263 ± 0.476	0.581
Dyslipidemia	-0.367 ± 0.583	0.529

Using the 4 coronary stenosis measurements as a patient-cluster response and cumulative logistic mixed-effects model, associations with the presence of an anomalous coronary, demographics, symptom and comorbidities were considered. The presence of an anomalous origin did not increase the likelihood of greater CAD severity. SE, Standard error; LMCA, Left main coronary artery; LAD, left anterior descending coronary artery; LCx, left circumflex; RCA, right coronary artery; CAD, coronary artery disease

**Table 5:** Odds ratio of each coronary having more severe stenosis based on the location of the anomalous coronary

Stenosis severity in:	Age	Gender	Anomalous LMCA	Anomalous LAD	Anomalous LCx	Anomalous RCA
Overall(clustered)	↑	0.6(0.3-1.0)	0.9(0.3–2.5)	-	0.5(0.1–1.5)	0.6(0.3–1.4)
LMCA	↑	0.5(0.2–1.3)	1.5(0.3–6.9)	-	-	0.6(0.2-2.0)
LAD	↑	0.6(0.3–1.1)	0.9(0.3-3.0)	-	0.8(0.2–2.7)	0.8(0.3-2.0)
LCx	↑	0.7(0.3–1.7)	0.8(0.2–3.4)	-	-	0.8(0.3–2.5)
RCA	↑	1.7(0.8–3.6)	1.0(0.2–3.7)	-	0.6(0.1-3.0)	1.0(0.3–2.7)

Odds ratios and 95% confidence intervals are shown for statistically significant correlations in multivariable modeling that adjusted for age, sex, comorbidities, and which coronary was anomalous. Blank cells indicate no statistical association. LMCA, Left main coronary artery; LAD, left anterior descending coronary artery; LCx, left circumflex; RCA, right coronary artery

### Outcomes

Among the 207 patients with anomalous coronary origin, 42 had a history of hospitalization due to cardiovascular diseases, while 10 were hospitalized specifically for angina. Additionally, cardiac procedures such as valve replacement, radiofrequency ablation, pacemaker installation, coronary artery bypass grafting, and stent implantation were performed on 26 patients. Furthermore, 6 patients required hospitalization for other reasons. Notably, coronary calcification on diagnosis was observed in 88 patients, while the remaining 119 patients exhibited no evidence of calcification at the time of AAOCA diagnosis. In addition, calcification was reported in 24 out of 26 patients with coronary artery disease, with 13 of them exhibiting calcification in coronary arteries of the anomalous origin.

## Discussion

In this study, we found a prevalence of AAOCA in patients referred for CTA to be 0.23% in the decade. Notably, 16 patients (0.02%) were identified with high-risk abnormalities. Among these patient with AAOCA, the RCA was found to be the most prevalent anomalous coronary artery, followed by the LMCA, multiple coronaries, LCx, and LAD. A comparative analysis revealed that patients with both AAOCA and CAD tended to be older males, experiencing a greater frequency of syncope and dizziness than those without CAD. Our multivariable modeling analysis indicated no association between AAOCA and the severity of CAD stenosis in the corresponding coronary.

Several publications have reported a prevalence of AAOCA ranging from 0.1 to 1.0% [1, 9, 12, 30, 31], which aligns with our findings based on CTA results. However, there are some discrepancies in other studies. For instance, Jiang et al. identified a prevalence of coronary artery anomalies as high as 11.12% in the southwest region of China [32]. This discrepancy can be attributed to two factors: different detection methods (angiography used in their study) and variations in the study populations (including patients with myocardial bridges and coronary artery aneurysms/ectasias). After excluding these cases, the prevalence was reduced to 0.7%. In a population-based study, the prevalence of coronary artery abnormalities detected by CTA was higher (2.6%) [33]. This difference may be attributed to our focus solely on anomalous coronary origin, which encompassed anomalies of anomalous origin, course, termination or anomalies of intrinsic coronary atrial anatomy in their study. Furthermore, ethnicity has been shown to influence the incidence of AAOCA, for example, Pan et al. reported an incidence of 3.93% among Uyghur patients compared to 2.34% among Han patients [34]. Similarly, Schmitt et al. reported an incidence of 2.5% (44 out of 1758 patients) [35], Duran et al. reported a rate of 5.79% (42 out of 725 patients) [36], and Shi et al. reported a rate of 6.6% (16 out of 242 patients) [37]. Our study also observed a higher prevalence of AAOCA in men compared to women, supporting previous findings from different studies [38].

Moreover, anomalous aortic origin of a coronary artery has been identified as a predominant cause of SCD in young individuals and athletes, potentially due to the impact of dilated aorta-pulmonary artery on coronary blood supply [12]. In patients with AAOCA, current U.S. and European guidelines recommend intervention in the presence of symptoms or evidence of ischemia [12, 13]. They strongly recommend intervention for all patients with the left coronary artery originating from the right sinus, or for patients with the right coronary artery originating from the left sinus who are symptomatic or experiencing coronary ischemia due to abnormal origin of the coronary artery [12, 13]. Additionally, the European guidelines use the high-risk features as a marker of prognosis and cardiovascular risk in these patients, including age, anatomy of coronary ostium and proximal coronary course, anomalous origin, exercise, and ischemia [12]. Evidence suggested that inter-arterial anomalous left coronary artery (ALCA) and anomalous right coronary artery (ARCA) are associated with an elevated risk of SCD, angina pectoris, myocardial infarction, or myocardial ischemia resulting from compression between vessels [12, 39, 40]. Cocco et al. found that the most common subtype is an ARCA arising from the left side followed by ALCA arising from the right side [11]. They also observed that SCD cases in young trained patients are primarily caused by ALCA while older patients exhibit equivalent or even higher incidence rates for ARCA-related SCD [11]. Chidyagwai et al. reported that although RCA patients have lower likelihoods of experiencing exercise-induced SCD, certain studies indicate increased incidence of ventricular fibrosis [23]. However, an observational study by Clark et al. found no association between AAOCA detected in CTA and sudden death [41]. Nevertheless, our study did not provide evidence of SCD case.

In this study, there are no association between the presence of anomalous origin and CAD stenosis severity in the corresponding coronary. Similarly, others reported that the presence of an anomalous vessel does not appear to increase the chances of CAD [28, 29]. The study conducted by Gräni et al. did not provide any evidence supporting a higher occurrence of obstructive or non-obstructive CAD among anomalous compared to non-anomalous coronary arteries [42]. Amado and colleagues concluded that none of the AAOCA types were associated with cardiac events [15], which aligns with our findings. In another study, there is no instances of cardiac death or adverse coronary events caused by the coronary anomaly during follow-up [43]. In addition, Jiang et al. demonstrated that anomalous origin does not increase the severity of CAD within anomalous coronaries among adults with AAOCA [38], while an anomalous LCx was associated with increased stenosis not only in itself but also in other coronaries. Similarly, Click et al. also shown that stenosis is significantly greater in an anomalous circumflex artery than in a non-anomalous circumflex artery in control subjects [24]. Several studies suggested that AAOCA may potentially lead to significant coronary stenosis [25, 44, 45]. Numerous mechanisms have been proposed to elucidate this association. For instance, some researcher have suggested that ischemic, blood flow perfusion, and hemodynamics may serve as potential mechanisms [14, 39]. Specifically, myocardial ischemia can be attributed to fixed extent component (e.g. slit-like ostium), proximal narrowing, acute take-off angle and intramural course with an elliptic vessel shape [16–19], as well as dynamic component (e.g., lateral compression) that are accentuated during exercise [12, 20–22]. In this study, coronary stenosis was mostly found in the middle or distal segments of coronary arteries. One patient was identified the plaque at the ostia, suggesting that AAOCA might be partially involved in the development of stenosis. The mechanisms of AAOCA related stenosis might be associated with angulation, ostial hypoplasia and hemodynamic abnormalities. For the patients with middle and distal coronary stenosis with AAOCA, the average age of 58.7 ± 11.6 years. We speculated that the stenosis might be more probably attributed to atherosclerosis. Despite knowledge about unique anatomical features of AAOCA like an acute angle of origin from the aorta or ostial slit possibly correlating with CAD development, consensus regarding underlying mechanisms leading to CAD remains elusive, thus further exploration is warranted.

### Limitations

However, there are several limitations that need to be considered. Firstly, this is a retrospective, single-center, and observational study focused on Chinese adult patients with AAOCA who were clinically referred for CTA. Therefore, the prevalence reported in this study is solely applicable to this specific population and may not be generalized to the other groups. Secondly, this study lacked granularity of the anomalous coronaries details, including coronary anatomy and course. Therefore, we did not further classify the trajectories of anomalous coronary arteries into various courses, such as inter-arterial, retro-aortic, trans-septal, retro-cardiac or pre-pulmonic courses. Thirdly, due to the lack of prospective follow-up, the Kaplan-Meier curve analysis and the hazard ratios for outcomes were not feasible as they require time variables. Additionally, there may have been missed events that occurred during the study period. Fourthly, it is noteworthy that the information gathered was either based on patient-reported surgical history or surgical records available within the hospital system. Therefore, it remains uncertain whether the surgeries were specifically performed due to AAOCA. Finally, we only compared the CAD within patients diagnosed with AAOCA and lacked matched patients without AAOCA as true controls.

## Conclusions

In summary, AAOCA represents a rare congenital malformation of coronary artery, as observed in adults undergoing CTA and does not appear to contribute to increased obstructive coronary artery disease. This finding might help for the management of patients with AAOCA.

### Electronic supplementary material

Below is the link to the electronic supplementary material.

Supplementary Material 1

## Data Availability

The datasets used and/or analysed during the current study are available from the corresponding author on reasonable request.

## Abbreviations

Aortic origin of coronary artery: AAOCA
Anomalous left coronary artery: ALCA
Anomalous right coronary artery: ARCA
Coronary artery disease: CAD
Computed tomographic angiography: CTA
Left anterior descending: LAD
Left circumflex: LCx
Left main coronary artery: LMCA
Right coronary artery: RCA
Sudden cardiac death: SCD
