# Supplementary material for: Anomalous origin of the coronary artery: prevalence and coronary artery disease in adults undergoing coronary tomographic angiography
Source: BMC Cardiovasc Disord. 2024 May 23;24:271. doi: 10.1186/s12872-024-03942-8 (PMC11112793; doi:10.1186/s12872-024-03942-8)
Supplement: Supplementary file 1 — Supplementary Material 1 [file 12872_2024_3942_MOESM1_ESM.docx]

**Supplementary table**

Table S1. Demographics, symptom and comorbidities stratified by presence and location of obstructive CAD

|  | All | Absent CAD | Only anomalous coronary with CAD | Normal-origin coronary with CAD | P value |
| --- | --- | --- | --- | --- | --- |
|  | N=207 | N=181 | N=6 | N=20 |  |
|  | n(%) | n(%) | n(%) | n(%) |  |
| Age, years | 58.7±11.6 | 58.0±11.6 | 66.8±9.9 | 61.9±11.2 | 0.090 |
| Male | 133(64.3) | 112(61.9) | 4(66.7) | 17(85.0) | 0.129 |
| Presenting symptom |  |  |  |  |  |
| Chest pain | 61 (29.5) | 52 (28.7) | 3 (50.0) | 6 (30.0) | 0.532 |
| Syncope | 4 (1.9) | 2 (1.1) | 0 (0) | 2 (10.0) | 0.022 |
| Dizziness | 9 (4.3) | 5 (2.8) | 0 (0) | 4 (20.0) | 0.001 |
| Palpitation | 15 (7.2) | 13 (7.2) | 0 (0) | 2 (10.0) | 0.708 |
| Dyspnea | 6 (2.9) | 6 (3.3) | 0 (0) | 0 (0) | 0.643 |
| Angina | 7 (3.4) | 5 (2.8) | 1 (16.7) | 1 (5.0) | 0.166 |
| Risk factors |  |  |  |  |  |
| Hypertension | 55 (26.6) | 45 (24.9) | 2 (33.3) | 8 (40) | 0.325 |
| Diabetes mellitus | 21 (10.1) | 16 (8.8) | 0 (0) | 5 (25.0) | 0.0542 |
| Dyslipidemia | 16 (7.7) | 12 (6.6) | 1 (16.7) | 3 (15.0) | 0.294 |

Baseline characteristics of 207 adult patients diagnosed with AAOCA are shown as counts (and relative frequencies) stratified by the presence and location. Obstructive coronary artery disease (CAD) was defined as≥50% stenosis in the left main coronary artery (LMCA), the right coronary artery (RCA), left anterior descending (LAD), or left circumflex (LCx) coronary artery. The frequencies of patients with AAOCA affecting each of the 4 or multiple coronaries were stratified based on the presence and location of any CAD. P value resulted from χ^2^ tests comparing patients with no CAD, CAD in only the anomalous coronary, and CAD in normal-origin coronaries. AAOCA, anomalous aortic origin of a coronary artery.

Table S2. Factors associated with greater severity of LMCA stenosis

|  | Coefficient±SE | P value |
| --- | --- | --- |
| Anomalous coronary: LMCA | 0.524±0.877 | 0.550 |
| Anomalous coronary: LAD | -16.026±0.000 | - |
| Anomalous coronary: LCX | -16.404±3066.391 | 0.996 |
| Anomalous coronary: RCA | -0.598±0.745 | 0.423 |
| Age, years | 0.060±0.027 | 0.023 |
| Gender: male | 0.622±0.603 | 0.303 |
| Chest pain | -1.341±0.903 | 0.138 |
| Syncope | 1.194±1.464 | 0.415 |
| Dizziness | 1.376±0.905 | 0.128 |
| Palpitation | -0.283±1.132 | 0.803 |
| Dyspnea | 2.608±1.445 | 0.071 |
| Angina | 1.228±1.218 | 0.314 |
| Hypertension | 0.571±0.593 | 0.335 |
| Diabetes mellitus | 0.564±0.717 | 0.432 |
| Dyslipidemia | -1.132±1.040 | 0.277 |

Age was associated with a greater grade of LMCA stenosis. The presence of an anomalous LMCA had no association with the CAD severity in the LMCA. SE, Standard error; LMCA, Left main coronary artery; LAD, left anterior descending coronary artery; LCx, left circumflex; RCA, right coronary artery; CAD, coronary artery disease.

Table S3. Factors associated with greater severity of LAD stenosis

|  | Coefficient±SE | P value |
| --- | --- | --- |
| Anomalous coronary: LMCA | 0.192±0.651 | 0.768 |
| Anomalous coronary: LAD | -17.029±0.000 | - |
| Anomalous coronary: LCX | 0.484±0.704 | 0.491 |
| Anomalous coronary: RCA | 0.127±0.516 | 0.805 |
| Age, years | 0.068±0.016 | <0.000 |
| Gender: male | 0.166±0.347 | 0.632 |
| Chest pain | 0.340±0.382 | 0.374 |
| Syncope | 0.137±1.105 | 0.901 |
| Dizziness | 0.737±0.718 | 0.305 |
| Palpitation | -0.479±0.682 | 0.482 |
| Dyspnea | -0.201±1.175 | 0.864 |
| Angina | 0.539±0.786 | 0.493 |
| Hypertension | 0.080±0.372 | 0.830 |
| Diabetes mellitus | 0.140±0.516 | 0.786 |
| Dyslipidemia | -0.077±0.614 | 0.900 |

Age was significantly associated with a greater grade of LAD stenosis. The presence of an anomalous LAD had no association with the CAD severity in the LAD. SE, Standard error; LMCA, Left main coronary artery; LAD, left anterior descending coronary artery; LCx, left circumflex; RCA, right coronary artery; CAD, coronary artery disease.

Table S4. Factors associated with greater severity of LCx stenosis

|  | Coefficient±SE | P value |
| --- | --- | --- |
| Anomalous coronary: LMCA | -0.695±0.953 | 0.466 |
| Anomalous coronary: LAD | -15.730±0.000 | - |
| Anomalous coronary: LCX | -16.314±2617.550 | 0.995 |
| Anomalous coronary: RCA | -0.027±0.693 | 0.969 |
| Age, years | 0.051±0.025 | 0.040 |
| Gender: male | 0.330±0.540 | 0.541 |
| Chest pain | -1.383±0.785 | 0.078 |
| Syncope | 0.953±1.416 | 0.501 |
| Dizziness | 2.204±0.824 | 0.007 |
| Palpitation | -3.289±1.736 | 0.058 |
| Dyspnea | -15.005±5036.181 | 0.998 |
| Angina | -0.172±1.236 | 0.889 |
| Hypertension | 0.866±0.532 | 0.104 |
| Diabetes mellitus | -0.180±0.770 | 0.816 |
| Dyslipidemia | -2.059±1.036 | 0.047 |

Age and dizziness were associated with a greater grade of LCx stenosis. The presence of an anomalous LCx had no association with the CAD severity in the LCx. SE, Standard error; LMCA, Left main coronary artery; LAD, left anterior descending coronary artery; LCx, left circumflex; RCA, right coronary artery; CAD, coronary artery disease.

Table S5. Factors associated with greater severity of RCA stenosis

|  | Coefficient±SE | P value |
| --- | --- | --- |
| Anomalous coronary: LMCA | -0.540±0.831 | 0.516 |
| Anomalous coronary: LAD | -16.340±0.000 | - |
| Anomalous coronary: LCX | 0.390±0.886 | 0.660 |
| Anomalous coronary: RCA | 0.052±0.619 | 0.933 |
| Age, years | 0.037±0.019 | 0.054 |
| Gender: male | 0.293±0.442 | 0.507 |
| Chest pain | -0.752±0.531 | 0.157 |
| Syncope | 0.093±1.283 | 0.943 |
| Dizziness | 1.473±0.753 | 0.051 |
| Palpitation | -0.393±0.907 | 0.664 |
| Dyspnea | -16.056±4550.107 | 0.9987 |
| Angina | 1.618±0.804 | 0.044 |
| Hypertension | 0.413±0.445 | 0.353 |
| Diabetes mellitus | 0.016±0.625 | 0.980 |
| Dyslipidemia | -0.971±0.853 | 0.255 |

Angina was associated with a greater grade of RCA stenosis. The presence of an anomalous RCA had no association with the CAD severity in the RCA. SE, Standard error; LMCA, Left main coronary artery; LAD, left anterior descending coronary artery; LCx, left circumflex; RCA, right coronary artery; CAD, coronary artery disease.
